# Understanding the genetics behind complex human disease with large-scale iPSC collections

**DOI:** 10.1186/s13059-017-1276-1

**Published:** 2017-07-20

**Authors:** Amanda E. Yamasaki, Athanasia D. Panopoulos, Juan Carlos Izpisua Belmonte

**Affiliations:** 1 Department of Biological Sciences, University of Notre Dame, Notre Dame, IN 46556 USA; 2 Gene Expression Laboratory, Salk Institute for Biological Studies, La Jolla, CA 92037 USA

## Abstract

Three recent studies analyzing large-scale collections of human induced pluripotent stem cell lines provide valuable insight into how genetic regulatory variation affects cellular and molecular traits.

Induced pluripotent stem cells (iPSCs) have been widely used as an important model system for human development and disease. They provide a potentially unlimited cell source for regenerative medicine, as well as a system in which to model patient-specific disease and test drug toxicity and effectiveness [1–3]. However, individual iPSC lines have been shown to be heterogeneous, raising questions about the suitability of iPSCs for genetic studies [4]. While prior studies have begun to address these concerns, they have thus far been performed with limited numbers of individuals, identifying only genetic variants that exert strong effects on phenotype, such as those underlying Mendelian traits [1–3].

Now, three recent studies have undertaken large-scale genomic analysis of iPSCs from hundreds of individuals (ranging between approximately 100 and 300 individuals) and all iPSC lines and accompanying data are available to the scientific community [5–7]. These studies have demonstrated that iPSCs are a valuable model system to study the function of genetic variation. Each has provided significant insight into the role of both rare and recurrent single-nucleotide polymorphisms and copy number variations (CNVs) in iPSCs and their phenotypic consequences. Furthermore, through comprehensive mapping of expression quantitative trait loci (eQTL), they illustrate the power of iPSCs to determine the functions of genetic variants in normal human phenotypic variation.

The combinatorial approaches utilized by each of these three studies have the distinct benefit of being able to correlate specific genotypes to variations in gene expression levels and provide a resource that allows the prediction of the consequences of genetic changes on phenotype variation [8]. All three studies mapped eQTLs for iPSCs, identifying regions of variation that associate with changes in mRNA expression. They also describe causal common variants for iPSC-specific eQTL genes, suggesting that iPSCs have a distinct regulatory landscape [5–7]. DeBoever et al. [6] report CNVs eQTLs in intergenic regions that can affect gene expression, and Carcamo-Orive et al. [5] demonstrate that Polycomb target genes can contribute significantly to variability, suggesting that heterogeneity in iPSCs can also be independent of genetics. By performing these types of combinatorial genomic analyses on large cohorts, these studies have provided novel insight into the functions of genetic variants in iPSCs.

The data provided in these three large-scale studies represent the highest resolution map of common regulatory variations in human iPSCs. Since Kilpinen et al. [7] and Carcamo-Orive et al. [5] analyzed multiple iPSC clones for each individual, they were able to demonstrate that genetic background effects exert a larger influence on variation in resultant iPSC lines than any other non-genetic factor, including copy number status, culture conditions, passage, and gender. This seems to indicate that for systematically generated lines the majority of iPSC heterogeneity is driven by inherent genetic variation between individuals, rather than by any effects of culture duration or conditions, or of the reprogramming process itself. However, Kilpinen et al. [7] also identified recurrent genetic abnormalities in iPSC lines as well as possible variations that may be conferring a selective advantage, and all three reports further showed that a large proportion of genomic variations between iPSC lines affected genes involved in stem cell maintenance, and the efficiency with which iPSCs differentiate [5–7]. It is possible that this variation could affect expression of these genes, and thus the pluripotency or differentiation capabilities of these cells. Further studies are needed to determine whether these genetic variants could affect the current gene-expression based methods of evaluating iPSC pluripotency and differentiation efficiency, or whether these effects are so small that they are entirely outweighed by environmental factors [6].

## How does understanding genomic variation in iPSCs help in the study of human disease?

The correlations between genomic variation and functional consequences are of particular interest in iPSCs. Since these cells can theoretically be differentiated into any cell type, they allow for the analysis of specific genomic changes that may have significantly different effects dependent on cell type. For example, Kilpinen et al. [7] identify a genomic variation in iPSCs that affects the regulation of *TERT* expression and telomerase activity, which they showed has significant effects in pluripotent or stem-like cells, but likely exerts little effect in differentiated cells, where *TERT* expression is usually silenced. However, cancer cells reactivate telomerase activity, meaning that this particular genomic variant, and others like it, may be useful to study diseases that affect cells only in limited states of cell growth and differentiation [7]. DeBoever et al. [6] showed that rare inherited variants with moderate effect can also be examined in this model system. They found that rare single-nucleotide variants (SNVs) in iPSC lines generally act to decrease expression of their associated genes, but exert a much smaller effect than rare CNVs, despite being more abundant [6]. These rare variants were not previously detectable in studies using smaller sample sizes. Some of these rare SNVs and CNVs occur in disease-associated loci and were more likely than common variants to have established roles in disease [6], but are difficult to examine using large-scale human cellular model approaches that would require large numbers of difficult to obtain and/or rare cell types. iPSCs could instead be used to generate a theoretically limitless population of cells that could be differentiated into the relevant cell types and used to study the effects of these rare variants on cellular phenotype and function, or combined with gene-editing technology to determine the mechanism behind the effects of the variant [3]. Thus, these resources are not limited to analysis of pluripotency, but can also serve as powerful tools for a range of questions related to development or disease.

## Conclusions and future work

Overall, these collective findings provide a valuable resource for understanding the genomic and phenotypic variation in iPSCs, and the drivers of this variation that are directly relevant to the use of these cells in understanding disease. This work serves as an important foundation for utilizing iPSCs to test variants identified by genome-wide association studies, as iPSCs can be readily used to interrogate variations that have functional consequences which may be driving disease phenotypes [9]. In addition, predicted models for regulatory networks can be tested using large databases of genomic data [8]. For instance, Carcamo-Orive et al. [5] utilize the data generated in their study to identify seven genes that serve as key drivers for the genomic variability in iPSCs. The use of iPSCs enables the distinct advantage of not being limited to analysis of molecular phenotypes, but also physiological phenotypes relevant to disease [6]. These large-scale genetic analyses can be used to dissect complex diseases and specific drug–genotype interactions [6], even in cases where variants have no effect on the normal function of a gene product, or indeed on the disease phenotype, but are highly relevant to the patient-specific response to treatment [10]. For example, some genetic variants may be unassociated with known diseases, but could still have an effect on individual responses to drug treatment, such as those based on alterations in immunological or metabolic processing [10]. These genetic variants may be rare, only detectable by screening hundreds or thousands of cell lines. Study of rare variants can be advanced by utilizing iPSC data made available by studies like these, or by generating new lines that can be stored, cultured, and differentiated into any relevant cell type without the need for invasive or repeated sample collection from patients. Thus, the knowledge gained by large-scale genomic studies of iPSCs has broad implications that extend beyond the stem cell field.

## Abbreviations

CNV: Copy number variation
eQTL: Expression quantitative trait locus
iPSC: Induced pluripotent stem cell
SNV: Single-nucleotide variant
